# 

**Published:** 1875-06

**Authors:** 


### Table of Contents.

### ORIGINAL COMMUNICATIONS.

Uterine Displacements treated by Position, Pneumatic Pressure and Mechanical Appliances. *By Prof. H. F. Campbell, of Augusta, Ga.*, 129

Clinical Studies with Non-Nauseating Doses of Ipecacuanha, chiefly in Intermittents. *By Alfred A. Woodhull, M. D., Assistant Surgeon U. S. Army* .... 136

Fistula of the Cornea and its Treatment. *By A. W. Calhoun, M. D.* 146

Errors of Diagnosis. *By A. Couvert, M. D.* .... 150

Cantharidal Stranguary. *By L. E. Starr, M. D., of Bibb county, Ala.* .. 152

Ovariotomy. *By A. Couvert, M. D.* .... 153

Quinine in Uterine Conception: *By J. S. Wetherly, M. D., of Montgomery, Ala.* .... 182

### ATLANTA ACADEMY OF MEDICINE.

Atrophy of the Optic Nerve, treatment by Electricity—Removal of the Eyeball for Sympathetic Ophthalmia, 154—Detachment of the Retina, treatment, cure, 157—Large Ovarian Multiple cyst, complicating pregnancy, tapping, subsequent premature labor and death, probably by rupture of the cyst, 159—Collins' Antidote for Opium—Carbazotate of Ammonia—Salicylic Acid—Rupture of Perineum, operation, secondary hemorrhage, 161—Cystic Tumor of the Abdominal Wall in negro woman—Multiple Ovarian Cyst in a young lady of sixteen, 164—Best time for operating Ovarian Cysts, 165—Eneucleation of the Eyeball for Sympathetic Ophthalmia, 156—Cerebro-Spinal Meningitis, 168—Fistula of the Cornea, new method of treatment—Cutting Short Gonorrhœa, 168—Gonorrhœa in young infant, 170—Congenital Absence of Anus, feces pass urethra, 171.

### OUR EXCHANGES.

Proceedings of the American Medical Association.... 171

Pat's Criticism.... 186

Dr. Bolling's Address at Louisville.... 187

### BIBLIOGRAPHICAL.

Pamphlets .... 187

### EDITORIAL.

The American Medical Association.... 189

Medical Association of Alabama....

Prize Essay .... 191

